# Exercise duration: Independent effects on acute physiologic responses and the need for an individualized prescription

**DOI:** 10.14814/phy2.15168

**Published:** 2022-02-10

**Authors:** Gerhard Tschakert, Tanja Handl, Lena Weiner, Philipp Birnbaumer, Alexander Mueller, Werner Groeschl, Peter Hofmann

**Affiliations:** ^1^ Institute of Human Movement Science, Sport & Health University of Graz Graz Austria

**Keywords:** distinct duration phases, duration thresholds, individualized prescription, maximal exercise duration

## Abstract

An individualization of exercise prescription is implemented mainly in terms of intensity but not for duration. To survey the need for an individualized exercise duration prescription, we investigated acute physiologic responses during constant‐load exercise of maximal duration (*t*_max_) and determined so‐called duration thresholds. Differences between absolute (min) and relative terms (% *t*_max_) of exercise duration were analyzed. Healthy young and trained male and female participants (*n* = 11) performed an incremental exercise test and one *t*_max_ constant‐load exercise test at a target intensity of 10% of maximal power output below the second lactate turn point (LTP_2_). Blood lactate, heart rate, and spirometric data were measured during all tests. *t*_max_ was markedly different across subjects (69.6 ± 14.8 min; range: 40–90 min). However, distinct duration phases separated by duration thresholds (DTh) were found in most measured variables. These duration thresholds (except DTh1) were significantly related to *t*_max_ (DTh2: *r* ^2^ = 0.90, *p* < 0.0001; DTh3: *r* ^2^ = 0.98, *p* < 0.0001) and showed substantial interindividual differences if expressed in absolute terms (DTh2: 24.8 ± 6.0 min; DTh3: 47.4 ± 10.6 min) but not in relative terms (DTh2: 35.4 ± 2.7% *t*_max_; DTh3: 67.9 ± 2.4% *t*_max_). Our data showed that (1) maximal duration was individually different despite the same relative intensity, (2) duration thresholds that were related to *t*_max_ could be determined in most measured variables, and (3) duration thresholds were comparable between subjects if expressed in relative terms. We therefore conclude that duration needs to be concerned as an independent variable of exercise prescription.

## Graphical abstract

To survey the need for an individualized exercise duration prescription, we investigated acute physiological responses during constant load exercise of maximal duration (*t*_max_), and determined so‐called duration thresholds (DTh). *t*_max_ was markedly different across subjects, and duration thresholds (except DTh1) were significantly related to *t*_max_ and showed substantial inter‐individual differences if expressed in absolute terms but not in relative terms. We therefore conclude that duration needs to be concerned as an independent variable of exercise prescription.

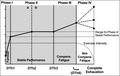

**New and Noteworthy**

To our knowledge, these are the first original data with respect to endurance exercise which show distinct duration phases and corresponding duration thresholds during maximal duration constant‐load exercise. These duration phases are characterized by specific acute physiologic responses and are suggested to be linked to distinct recovery times and training adaptations. Interestingly, the relative duration (% *t*_max_) at the thresholds are comparable across subjects although the maximal duration shows substantially interindividual differences.

## 1 INTRODUCTION

Exercise prescription is important to regulate exercise training in health and disease; however, usual prescription methods have been discussed critically (Iannetta et al., 2020). As a standard, we may actually suggest the so‐called FITT principle that prescribes training workload by frequency, intensity, time (duration/volume), and type (Burnet et al., 2019) as well as expanded versions of this principle (Reid et al., 2019). Currently, the main variable of exercise prescription is mostly intensity, although the duration may also have a major and independent impact on the grade of homeostatic disturbance during and after exercise, recovery time, and adaptation (Borsheim & Bahr, 2003; Milesis et al., 1976; Moghetti et al., 2016; Platonov, 1999; Viru, 1995; Viru et al., 1996). In a review article, Wenger and Bell (1986) nicely presented the effect of exercise duration on improvements of maximal oxygen uptake (V̇O_2max_) independent of exercise intensity, frequency, and length of the intervention period.

Therefore, an individualized exercise prescription needs to be applied with respect to all variables. Although important in endurance sports practice, this holds true even more for scientific studies (Hofmann & Tschakert, 2010) to set a solid basis for a comparison of studies and meta‐analyses. In a recent overview, we presented some theoretical basics regarding this question. However, experimental data on the impact of exercise duration as a single and independent variable are still missing (Hofmann & Tschakert, 2017).

Tremblay et al. (2005) critically mentioned that only a little research tempting to isolate the effects of exercise duration has been done. Some studies showed an exercise duration dependency for manifold physiologic effects during and/or after exercise such as muscular micro RNA release that controls posttranscriptional gene expression (Ramos et al., 2018), hormone release (Tremblay et al., 2005; Viru, 1995; Viru et al., 1996), hemodynamic and arterial elasticity and total vascular resistance (Karabulut et al., 2020), nocturnal heart rate (HR) and heart rate variability (HRV) (Myllymäki et al., 2012), excess postexercise oxygen consumption and metabolism (Bahr et al., 1987; Chad & Wenger, 1985, 1988; Gore & Withers, 1990a,b; Sedlock et al., 1989), as well as immune response (Diment et al., 2015).

However, detailed duration‐dependent differences for acute effects and chronic adaptations are still unclear. In the aforementioned studies, exercise duration was prescribed arbitrarily by means of fixed absolute values (e.g., 30, 45, 60, 90 min) but not on an individual basis. Regarding exercise intensity, standardized and well‐accepted individual markers, such as the first and second turn points for lactate (LTP_1_, LTP_2_) or ventilation (VT_1_, VT_2_), and the maximum power output (*P*_max_), have been prescribed in detail (Binder et al., 2008) and used for intensity prescription. In contrast, from the literature, it is obvious that for exercise duration, we still have a lack of information regarding relevant individual markers such as maximum duration or possible distinct duration domains. In addition, consistent models for an individualized prescription of endurance exercise duration are extremely rare and still rather theoretical (Hofmann & Tschakert, 2017). Important to notice is the fact that the maximum duration (*t*_max_) at given intensities, which may be called the maximal endurance capacity (Brooks et al., 2005, p. 495), can markedly differ across individuals with various (Mezzani et al., 2010) or even similar aerobic performance. As a consequence, prescribing fixed exercise durations with no regard to the individual *t*_max_ may result in heterogeneous acute responses and subsequent adaptations (despite the same relative intensity), which may explain at least in part the so‐called “nonresponder” phenomenon (Lin et al., 2021; Ross et al., 2015).

**FIGURE 1:**
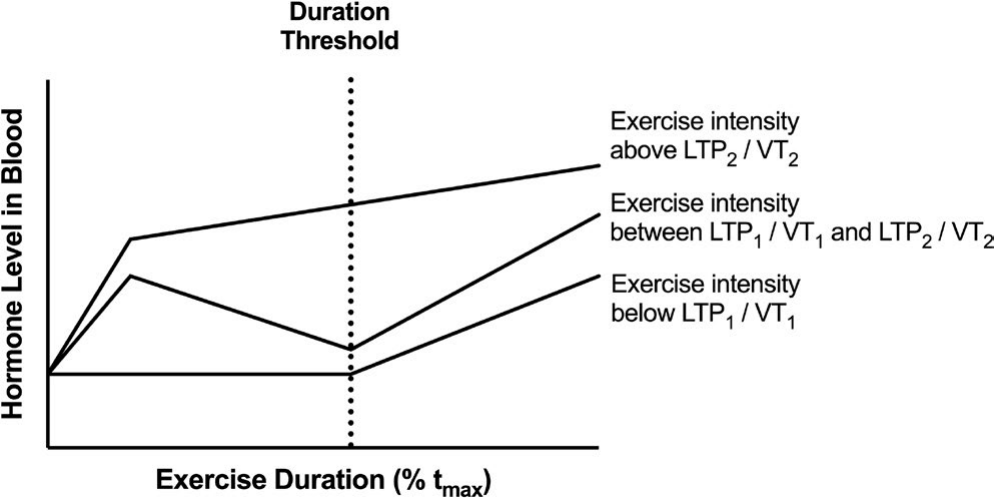
Schematic chart of the duration threshold concept. The duration threshold had to be exceeded in order to even induce hormonal responses (for intensities below LTP_1_) or to trigger a second and stronger endocrine activation (for intensities between LTP_1_ and LTP_2_). The high‐intensity exercise above LTP_2_ resulted in a further increase of hormonal responses when the duration threshold was exceeded (Viru, 1995, modified). *t*_max_, maximal time; LTP_1_, first lactate turn point; LTP_2_, second lactate turn point; VT_1_, first ventilatory threshold; VT_2_, second ventilatory threshold

It is obvious that the grade of homeostatic disturbance is higher if defined exercise intensities are sustained for the maximal duration (*t*_max_) than just for a certain percentage of *t*_max_. The individual duration‐dependent grade of homeostatic disturbance may be reflected by different recovery kinetics after exercise (Bahr et al., 1987; Chad & Wenger, 1985, 1988; Gore & Withers, 1990a,b; Sedlock et al., 1989) but even more by acute changes of physiologic responses during exercise (Viru, 1995; Viru et al., 1996). The moments when those changes occur represent essential individual markers of exercise duration according to the intensity markers LTP_1_, LTP_2_, and *P*_max_. Viru (1995) and Viru et al. (1996) emphasized the dependence of the magnitude of hormonal responses on exercise duration and described a so‐called *duration threshold* (DTh) determined for low and moderate but not for high exercise intensity (Figure 1). In line with this, Tremblay et al. (2005) suggested a duration threshold for hormonal responses particularly for low‐intensity exercise.

Hackney and Lane (2015) also investigated the endocrine reactivity to exercise and presented the “Hormonal Exercise Response Model” (HERM) with three interactive duration phases.

It can be assumed that these markers of exercise duration (phases and thresholds) are individual, different in time across subjects and that they strongly depend on the individual maximal duration at defined intensities.

A detailed theoretical concept was presented by Platonov (1999) who differentiated low, moderate, submaximal, and maximal domains of load with respect to duration separated by duration thresholds (DTh). These duration domains were prescribed as the phase of familiarization (1), phase of stable performance with two subphases (2a and 2b) also separated by a DTh, phase of compensated fatigue (3), and phase of not‐compensated fatigue in which the given intensity cannot be sustained any more (4). These time domains were associated with a selective degree of acute homeostatic disturbance, required recovery time, and suggested adaptations (Platonov, 1999, p. 48–51, 162–163).

To our knowledge, this is the first concept for an individualized prescription of exercise duration presented recently as part of a comprehensive theoretical concept for exercise prescription (Hofmann & Tschakert, 2017). This structure of distinct duration phases is supposed to hold true for intensities below and at LTP_2_ but not above LTP_2_ (Viru, 1995; Viru et al., 1996) (Figure 1). However, several aspects remain unclear. Currently, we still do not know the validity of such duration domains as described by Platonov (1999) and on which experimental data they are based.

In a first attempt to answer these pending questions, we conducted a pilot study to investigate the acute physiologic responses during maximal voluntary duration (100% *t*_max_) constant‐load exercise at defined exercise intensity. The aims of this study were (1) to prove differences in *t*_max_ between subjects, (2) to verify the occurrence of distinct phases of exercise duration according to the concept of Platonov (1999) by means of individual curve analysis of different physiologic parameters, and (3) to compare the interindividual differences in times of duration thresholds (*t*_DTh_) expressed in absolute (min) vs. relative terms (% of *t*_max_).

Our main hypotheses were that (1) *t*_max_ is substantially different across subjects although working at the same relative exercise intensity and that (2) during *t*_max_ constant‐load exercise, distinct phases of exercise duration, characterized by certain acute physiologic responses, can be observed and duration thresholds can be determined.

## 2 MATERIALS AND METHODS

### 2.1 Subject characteristics

Eleven healthy, young male (m; 9) and female (f; 2) subjects (age: 26.5 ± 3.2 years, height: 1.77 ± 0.09 m, weight: 77.9 ± 11.3 kg, maximum oxygen uptake [V̇O_2max_]: 50.7 ± 6.9 ml/kg min) participated in this study. V̇O_2max_ indicated a moderately trained state of all subjects. The participants gave their written informed consent before conducting the first test, and the Ethics Committee of the local University approved this study design (EC decision number: 39/72/63 ex 2017/18).

### 2.2 Experimental design

At the beginning of the study, all participants performed an incremental exercise test (IET) to determine V̇O_2max_ and the maximum power output (*P*_max_) as well as the power output at the first and second turn point for lactate (LTP_1_, LTP_2_) and ventilation (VT_1_, VT_2_), respectively (Binder et al., 2008). *P*_max_ and P_LTP2_ were used for exercise intensity prescription for a subsequent constant‐load test (CLT). This CLT was performed for the maximum voluntary sustainable duration (*t*_max_) at a defined intensity which was set at the same relative intensity for each subject. The IET and the *t*_max_ CLT were interspersed by at least 2 days.

#### 2.2.1 Incremental exercise test (IET)

The IET started with a 3 min resting period and a 3 min warm‐up phase at 20 W (f) or 40 W (m). Subsequently, the workload was increased by 15 W (f) or 20 W (m)/min until subjective exhaustion was reached. The following cooldown phase (active recovery) at the same intensity as in the warm‐up phase lasted 3 min; then, the IET was finalized by a 3 min resting period (passive recovery). Workload increase was adapted to male and female subjects in order to guarantee the same duration of approximately 15 min of exercise in the IET.

#### 2.2.2 Maximum time constant‐load test (*t*_max_ CLT)

In the *t*_max_ CLT, the target workload had to be sustained as long as possible (*t*_max_). This target workload was set at an exercise intensity of 10% *P*_max_ below P_LTP2_ from IET which was between LTP_1_ and LTP_2_. When the prescribed exercise intensity could not be sustained anymore although strong verbal encouragement, *t*_max_ was accomplished and the test was terminated.

The CLT started with a 3 min resting period followed by a three‐stage warm‐up phase over 5 min. Then, the target workload phase was conducted until *t*_max_ was reached. Subsequently, a 3 min active recovery phase at 20 W (f) or 40 W (m) and a following 3 min passive recovery phase finalized the CLT. No food or fluids were supported during the test.

### 2.3 Measurements and analysis

All tests were performed on an electronically braked cycle ergometer (Monark Ergomedic 839 E, Monark, Sweden) in a standard laboratory with defined climate conditions set at 21°C. A fan was used for cooling during the CLT.

#### 2.3.1 Heart rate and gas exchange parameters

Heart rate (HR) (Polar S810i, Polar Electro, Finland) and spirometric parameters (ZAN 600, ZAN, Austria), such as breathing frequency (BF), tidal volume (V̇T), ventilation (V̇E), oxygen consumption (V̇O_2_), carbon dioxide production (V̇CO_2_), ventilatory equivalents for oxygen (EqO_2_) and carbon dioxide (EQCO_2_), end‐tidal pressures for oxygen (PETO_2_) and carbon dioxide (PETCO_2_), O_2_ pulse, and respiratory exchange ratio (RER), were measured continuously during all tests. Gas analyzers for O_2_ and CO_2_ as well as the turbine to measure ventilation were calibrated before each test using standard gases and a 1 l syringe according to the manufacturers’ guidelines.

#### 2.3.2 Blood lactate and glucose concentrations

Blood lactate (La) and glucose (Glu) concentrations were obtained from capillary blood samples taken from a hyperemized ear lobe during all tests. For IET, blood samples were taken during the resting period and at the end of the warm‐up phase, at the end of each workload step as well as at the end of active and passive recovery. During the CLT, blood samples were taken during the resting period, after 2, 4, 6, 8, 10, 15, 20, 25, 35, 45, 55, 65 min, etc. until *t*_max_ was reached, and at the end of active and passive recovery. Blood samples were used for the determination of La and Glu by means of a fully enzymatic‐amperometric method (Biosen S‐line, EKF‐Diagnostics, Germany).

#### 2.3.3 Additional measurements

Rating of perceived exertion (RPE) was ascertained by means of the BORG scale (6–20 points) at the same moments when blood samples were taken during the *t*_max_ CLT.

In addition, an electrocardiogram (ZAN 600, ZAN, Austria) and manually measured blood pressure (SunTech Cycle BP, USA) were obtained from each subject and supervised by an experienced physician during IET for safety reasons (data not shown).

#### 2.3.4 Data analysis

The first and second turn points for lactate and ventilation (LTP_1_, VT_1_ and LTP_2_, VT_2_) from the IET were assessed by means of a computer‐aided linear regression breakpoint method (ProSport, Graz, Austria) (Hofmann et al., 2001). For LTP_1_/VT_1_, the region of interest (ROI) was set consistently between the first load step and 65–70% of *P*_max_. For LTP_2_/VT_2_, the ROI was set between LTP_1_/VT_1_ and *P*_max_.

**FIGURE 2:**
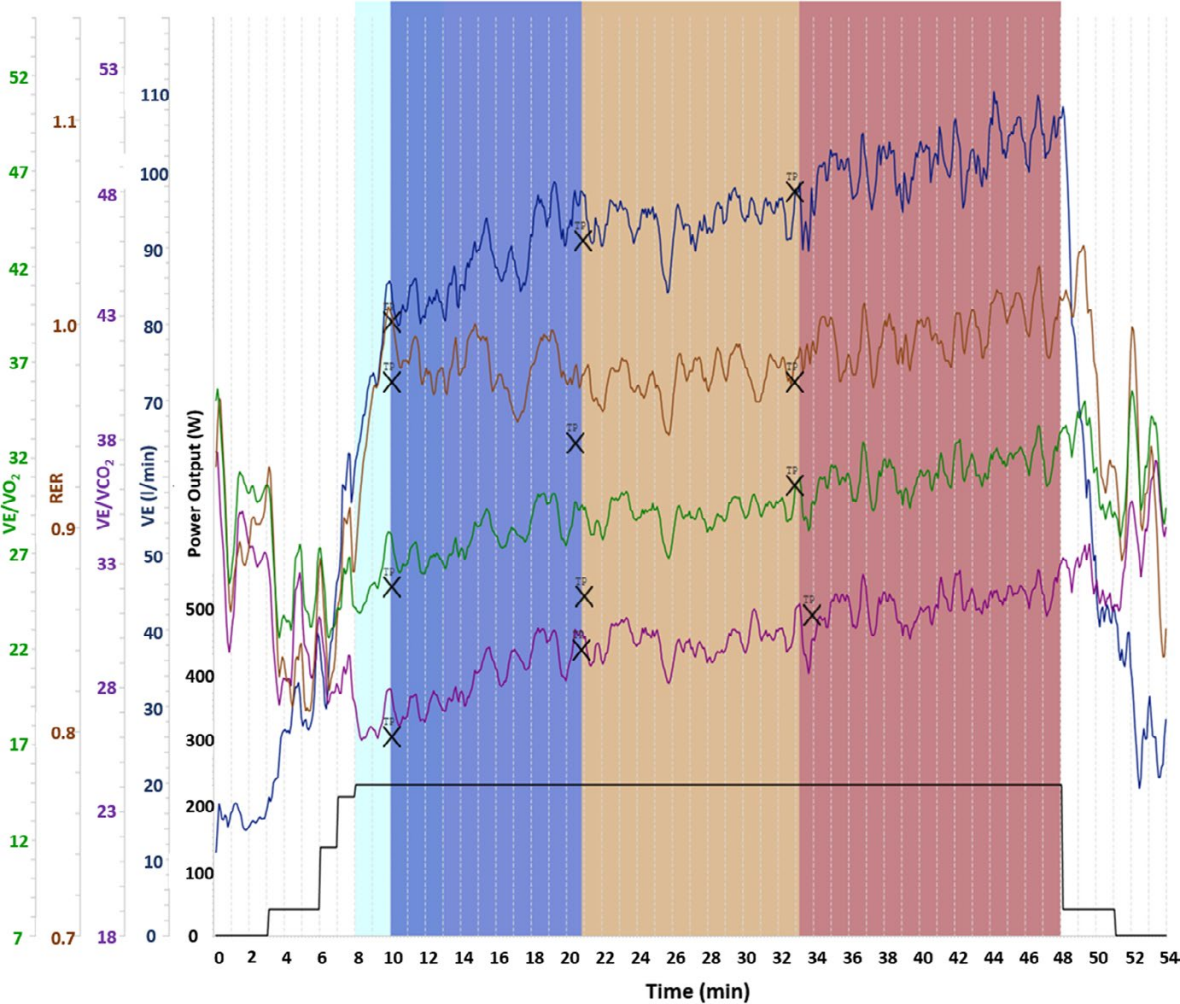
Time course and DTh determination for V̇E, V̇E/V̇O_2_, V̇E/V̇CO_2_, and RER during the *t*_max_ CLT. Data are individual values. DTh, duration threshold; V̇E, ventilation; V̇E/V̇O_2_, ventilatory equivalent for oxygen; V̇E/V̇CO_2_, ventilatory equivalent for carbon dioxide; RER, respiratory exchange ratio. Printout from the Vienna CPX Tool program

For the computer‐supported determination of the duration thresholds from the CLT, the software Vienna CPX Tool 1.0.0 (University of Vienna, Vienna, Austria) was used (Figure 2). A breakpoint regression method was applied to determine significant threshold‐like changes in variables with time. The ROI for the determination of each DTh was set between the two optically recognizable neighboring duration thresholds which were in accordance with the concept of Platonov (1999).

### 2.4 Statistics

All data are presented as means ± SD. For statistical analysis, GraphPad Prism 8.0.2 (GraphPad Software, Inc., USA) was used. All data were evaluated for normal distribution by means of the Kolmogorov‐Smirnov test and the Shapiro‐Wilk test. For the comparison of the slopes in consecutive duration phases, a one‐way repeated measures ANOVA with post‐hoc Sidak's multiple comparisons test was used for normally distributed data, and a Friedman Test with Dunn's multiple comparisons test was used for nonnormally distributed data. For other pairwise comparisons, significant differences were determined by dependent *t* test in case of normal data distribution. In case of no normal distribution, a Wilcoxon test was applied. Significant correlations were determined by linear regression. A level of significance was accepted to be *p* < 0.05.

## 3 RESULTS

### 3.1 Incremental exercise test (IET)

**FIGURE 3:**
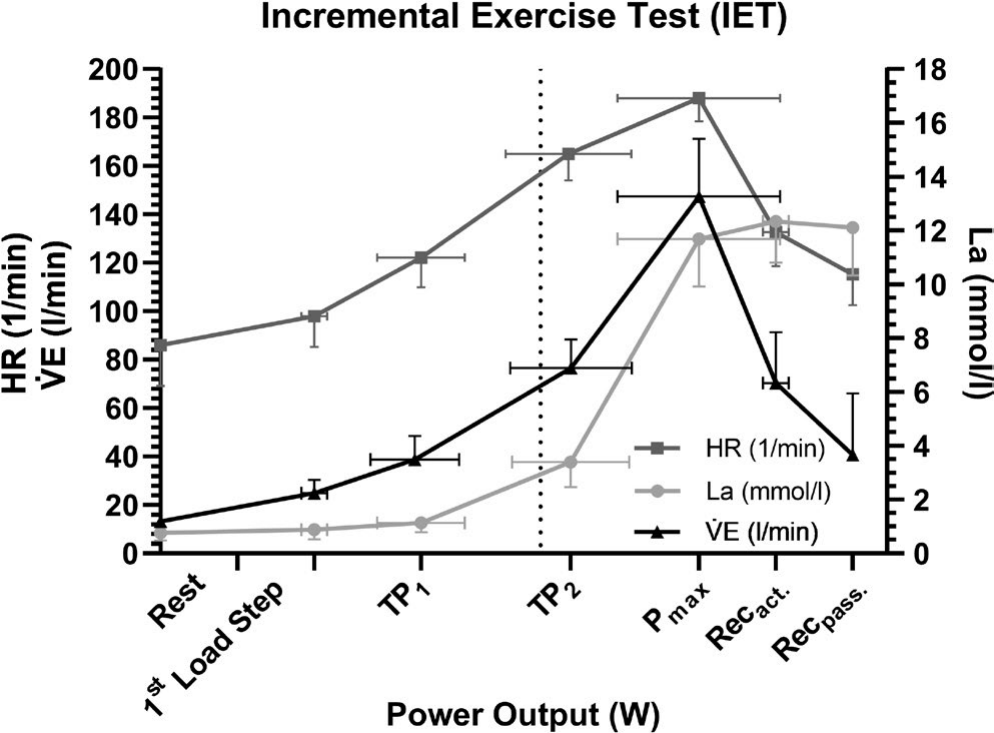
Time course for La, V̇E, and HR during the IET. Data are means and SD at rest, after 3 min of warm‐up, at the first (LTP_1_) and the second (LTP_2_) lactate turn point as well as at maximal power output and after 3 and 6 min of recovery. Data are shown by means and SD (*n* = 11). HR, heart rate; La, blood lactate concentration; V̇E, ventilation; TP_1_, first turn point; TP_2_, second turn point; *P*_max_, maximal power output; REC_act_, active recovery; REC_pass_, passive recovery

All subjects successfully performed the IET up to maximal voluntary exhaustion without any problems. Maximal exhaustion was verified by RER > 1.1 and reaching age‐predicted HR_max_. LTP_1_ and LTP_2_ were found at 35.8 ± 5.2% and 70.5 ± 2.1% of *P*_max_, respectively, and were not significantly different compared with VT_1_ (34.4 ± 6.7% *P*_max_) and VT_2_ (70.5 ± 2.5% *P*_max_) (*p* = 0.153 for LTP_1_ vs. VT_1_; *p* = 0.863 for LTP_2_ vs. VT_2_). Figure 3 shows the time course for mean ± SD for La, V̇E, and HR during the IET.

The intensity for *t*_max_ CLT was set at 10% *P*_max_ below LTP_2_ which was 194.6 ± 32.6 W representing 60.4 ± 2.2% of *P*_max_ and 73.3 ± 4.8% of V̇O_2max_.

### 3.2 *t*_max_ constant‐load test

#### 3.2.1 Interindividual differences in *t*_max_

Intensity prescription worked well and all physiologic responses in the *t*_max_ CLT such as for HR, V̇O_2_, and La were within narrow regions and comparable across subjects. A lactate steady state (LaSS) was found (at 3.59 ± 0.16 mmol/l) between 10 and 30 min during CLT for all participants according to the accepted LaSS definition (Beneke, 2003). *t*_max_ in the CLT showed no significant relationship to different markers of endurance performance (*P*_max_, V̇O_2max_, or *P*_LTP2_, for all *p* > 0.05). Importantly, *t*_max_ varied widely across subjects between 40 and 90 min despite the same relative workload and was found at 69.6 ± 14.8 min (Table 1).

**TABLE 1:** Slopes for V̇E, BF, EqO_2_, EqCO_2_, PETO_2_, PETCO_2_, and RPE within each duration phase (1, 2a, 2b, and 3) and the significance of the slope differences (*p* values) for 1 vs. 2a, 2a vs. 2b, and 2b vs. 3 (*n* = 11)

Variables	Phase 1 Rest–DTh1	Phase 2a DTh1–DTh2	Phase 2b DTh2–DTh3	Phase 3 DTh3–*t*_max_	*p* values 1 vs. 2a	*p* values 2a vs. 2b	*p* values 2b vs. 3
∆V̇E/*t*	21.22 ± 6.41	0.79 ± 0.40	0.17 ± 0.20	0.59 ± 0.43	<0.0001	0.0040	0.0133
∆BF/*t*	3.94 ± 2.07	0.43 ± 0.21	0.08 ± 0.16	0.43 ± 0.20	0.0397	0.0247	0.0397
∆EqO_2_/*t*	−1.32 ± 1.44	0.20 ± 0.07	0.02 ± 0.08	0.22 ± 0.12	0.0029	0.0193	0.0150
∆EqCO_2_/*t*	−1.66 ± 0.81	0.17 ± 0.09	0.04 ± 0.05	0.23 ± 0.14	<0.0001	0.0397	0.0089
∆PETO_2_/*t*	−3.89 ± 3.25	0.37 ± 0.23	0.05 ± 0.09	0.26 ± 0.16	<0.0001	0.0067	0.0499
∆PETCO_2_/*t*	3.48 ± 1.62	−0.23 ± 0.10	−0.09 ± 0.09	−0.19 ± 0.11	<0.0001	0.0148	0.0279
∆RPE/*t*	2.43 ± 1.10	0.11 ± 0.10	0.08 ± 0.06	0.16 ± 0.07	0.0002	0.8245	0.0499

Note

All presented variables showed significant slope differences for all comparisons except RPE for 2a vs. 2b.

Values are means and SD. The slope values for the single variables are presented as units of the variable per minute.

Abbreviations: BF, breathing frequency; DTh, duration threshold; EqCO_2_, ventilatory equivalent for carbon dioxide; EqO_2_, ventilatory equivalent for oxygen; PETCO_2_, end‐tidal pressure for carbon dioxide; PETO_2_, end‐tidal pressure for oxygen; RPE, rating of perceived exertion; *t*, time; *t*_max_, maximal time; V̇E, ventilation.

#### 3.2.2 Distinct duration domains

**FIGURE 4:**
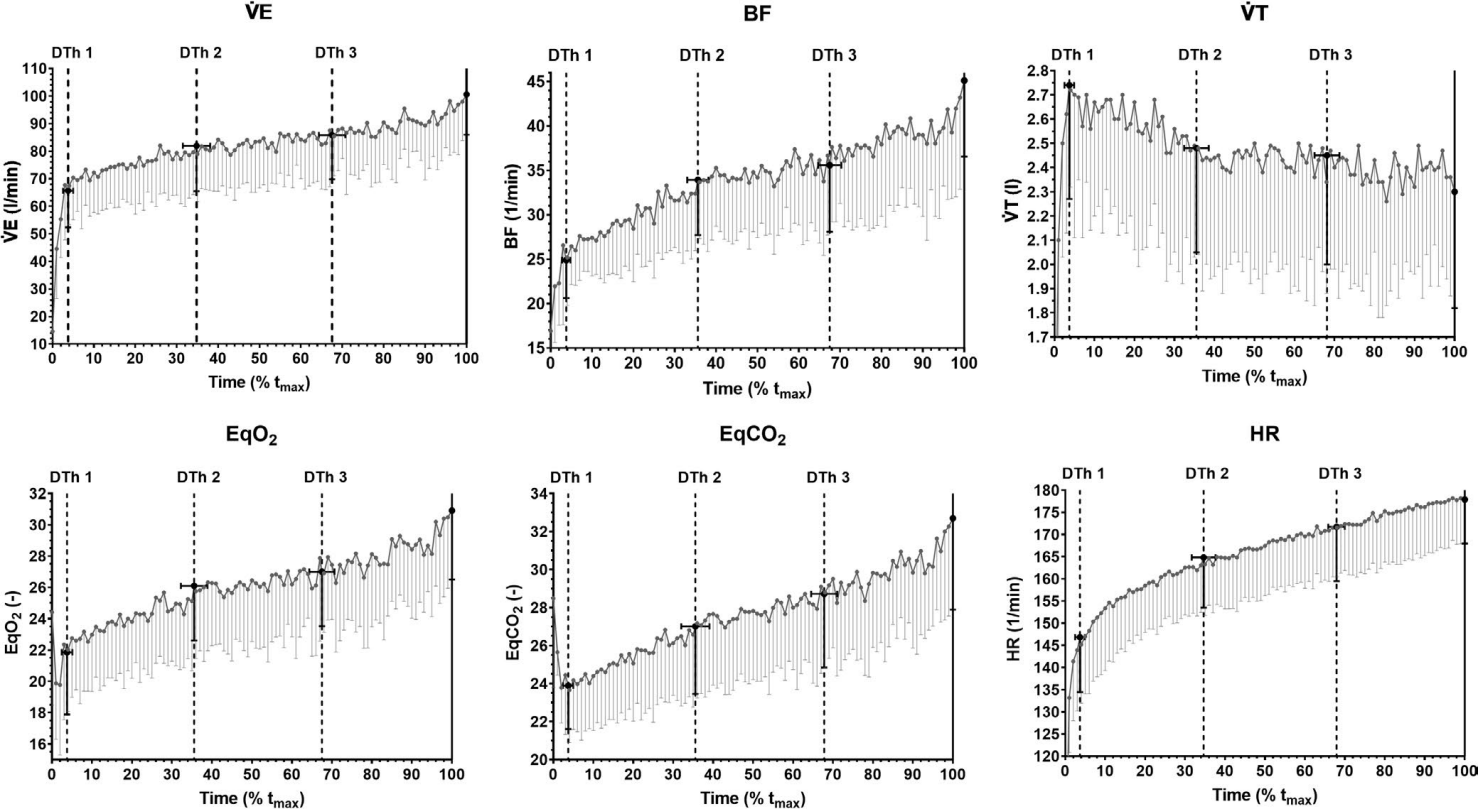
Averaged curve patterns (means and SD) during CLT showing distinct duration domains for V̇E, BF, V̇T, EqO_2_, EQCO_2_, and HR. Data are shown as means ± SD (*n* = 11). V̇E, ventilation; BF, breathing frequency; V̇T, tidal volume; EqO_2_, ventilatory equivalent for oxygen; EqCO_2_, ventilatory equivalent for carbon dioxide; HR, heart rate; DTh, duration threshold; *t*_max_, maximal time

**FIGURE 5:**
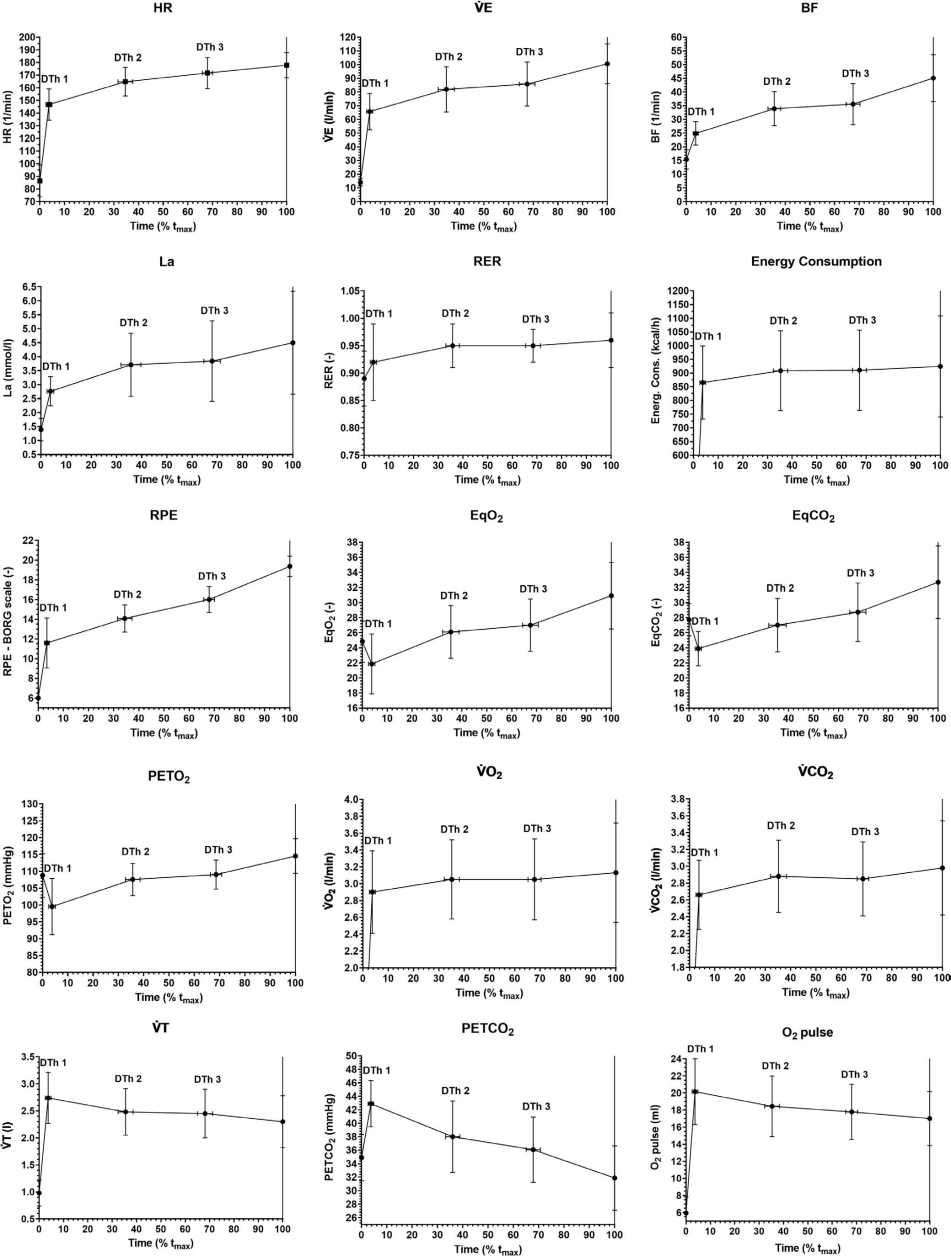
During the CLT, three duration thresholds (DTh1, DTh2, and DTh3) and according distinct duration domains (phases 1, 2a, 2b, and 3) were detectable for all parameters but not for Glu (not shown). Data are shown as means ± SD (*n* = 11). HR, heart rate; V̇E, ventilation; BF, breathing frequency, La, blood lactate concentration; RER, respiratory exchange ratio; RPE, rating of perceived exertion; EqO_2_, ventilatory equivalent for oxygen; EqCO_2_, ventilatory equivalent for carbon dioxide; PETO_2_, end‐tidal pressure for oxygen; V̇O_2_, oxygen consumption; V̇CO_2_, carbon dioxide production; V̇T, tidal volume; PETCO_2_, end‐tidal pressure for carbon dioxide; O_2_ Pulse, oxygen pulse; DTh, duration threshold; *t*_max_, maximal time

Three distinct duration domains could be observed in the CLT in individual as well as in averaged curve patterns for all parameters except blood glucose (Glu): (1) phase of familiarization (until DTh1), (2) phase of stable performance with two subphases 2a (until DTh2) and 2b (until DTh3), and (3) phase of compensated fatigue (until *t*_max_). As it is shown in Figures 4 and 5, the distinction of duration phases was not equally visible for each parameter (e.g., for HR). However, three duration thresholds were detected for all parameters at the same relative time except Glu.

Most parameters such as BF, V̇E, V̇O_2_, V̇CO_2_, EqO_2_, EQCO_2_, PETO_2_, RER, Energy Consumption, La and RPE showed a typical curve pattern as can be seen in Figure 5: phase 1, steep increase until DTh1 reaching values at target workload (*P*_target_) from IET; phase 2a, a slight increase above values at *P*_target_ from IET until DTh2; phase 2b, stable values slightly above values at *P*_target_ from IET until DTh3; and phase 3, increase again until *t*_max_. Because of the physiologic nature of the variables, V̇T, PETCO_2_, and O_2_ pulse showed a different pattern: (1) steep increase reaching values at *P*_target_ from IET, (2a) slight decrease below values at *P*_target_ from IET, (2b) stable values below values at *P*_target_ from IET, and (3) decrease again until *t*_max_.

Significant differences were found between the slopes for (1) rest–DTh1 (phase 1) vs. DTh1–DTh2 (phase 2a), (2) DTh1–DTh2 (phase 2a) vs. DTh2–DTh3 (phase 2b), and (3) DTh2–DTh3 (phase 2b) vs. DTh3–*t*_max_ (phase 3) for the variables presented in Table 1.

#### 3.2.3 Interindividual differences for duration thresholds (*t*_DTh_) and *t*_max_ in absolute vs. relative terms

**FIGURE 6:**
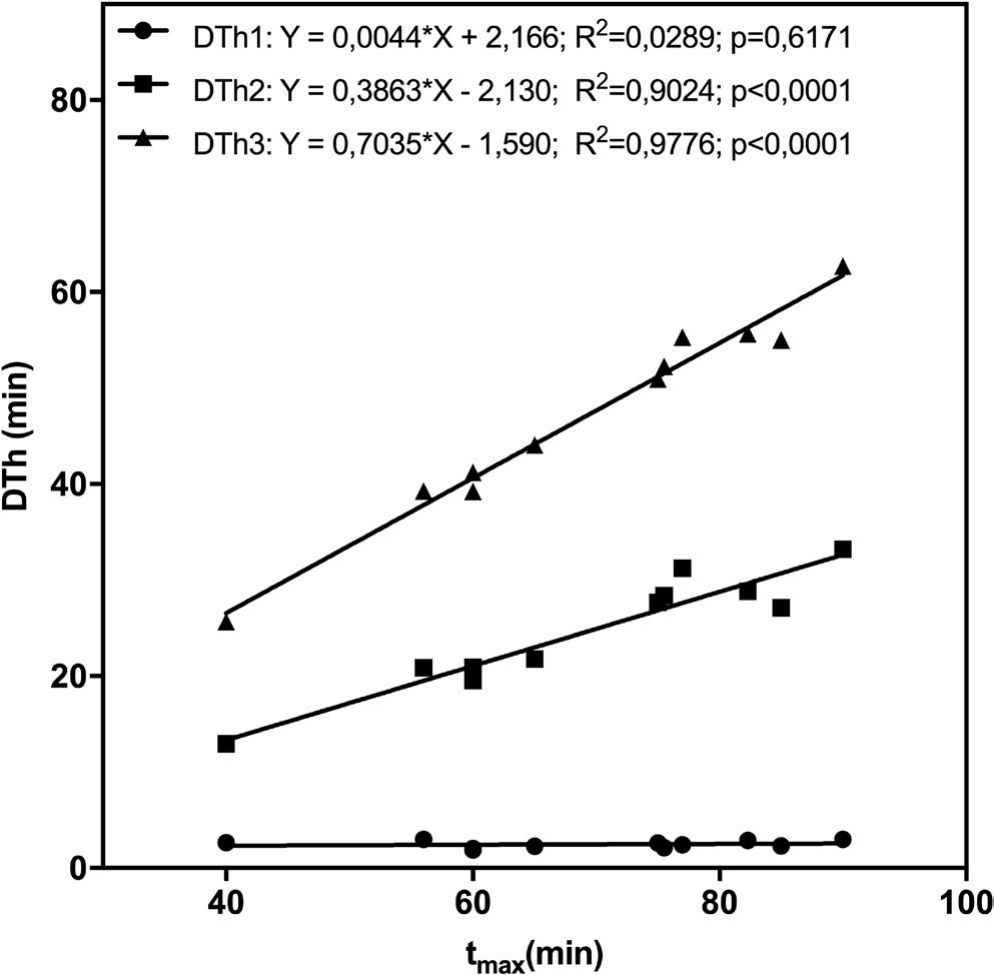
Correlation between duration thresholds and *t*_max_. DTh, duration threshold; *t*_max_, maximal time; *p*, significance value; *R* ^2^, coefficient of determination

As expected, time of DTh1, representing the end of the familiarization phase, was comparable across subjects but not significantly correlated to *t*_max_ (*r* ^2^ = 0.03, *p* = 0.62). In contrast, *t*_DTh2_ (*r* ^2^ = 0.90, *p* < 0.0001) and t_DTh3_ (*r* ^2^ = 0.98, *p* < 0.0001) showed a highly significant correlation to *t*_max_ (Figure 6).

Therefore, not only *t*_max_ but also *t*_DTh2_ and *t*_DTh3_ showed substantial interindividual differences if they were given in absolute terms (min) but not in relative terms (% *t*_max_) (Table 2). The interindividual SD values (expressed as % of the according means) for *t*_DTh2_ and *t*_DTh3_ were significantly smaller if DTh2 and DTh3 were given in relative terms (*t*_DTh2_: 8.6%; t_DTh3_: 4.1%) vs. absolute terms (t_DTh2_: 24.7%; t_DTh3_: 22.4%) (*p* < 0.0001). To show the robustness of the duration thresholds determination, the duration thresholds were averaged over 13 measurement parameters. The SD was marginal (Table 2). However, due to the small number of subjects (*n* = 11) and the high number of variables (*n* = 13), no conclusive statistics could be calculated.

**TABLE 2:** Times for DTh1, DTh2, DTh3, and *t*_max_ expressed in absolute (min) vs. relative terms (% *t*_max_) for the individual subjects and averaged overall subjects (*n* = 11)

Subjects	*t* abs. (min)			*t*_max_	*t* rel. (% *t*_max_)		
	DTh1	DTh2	DTh3		DTh1	DTh2	DTh3
	Mean ± SD	Mean ± SD	Mean ± SD		Mean ± SD	Mean ± SD	Mean ± SD
P 01^a^	2.11 ± 0.11	28.38 ± 0.66	52.27 ± 1.30	75.5	2.79 ± 0.15	37.60 ± 0.88	69.23 ± 1.73
P 02^a^	2.66 ± 0.12	12.92 ± 0.19	25.65 ± 0.55	40	6.65 ± 0.31	32.29 ± 0.48	64.13 ± 1.38
P 03^a^	1.90 ± 0.09	20.89 ± 1.18	41.19 ± 0.47	60	3.17 ± 0.15	34.82 ± 1.96	68.64 ± 0.78
P 04^a^	2.03 ± 0.11	19.53 ± 0.57	39.24 ± 1.31	60	3.38 ± 0.19	32.54 ± 0.96	65.40 ± 2.19
P 05^a^	2.42 ± 0.12	31.24 ± 1.16	55.31 ± 0.55	77	3.15 ± 0.16	40.57 ± 1.51	71.84 ± 0.71
P 06^a^	2.62 ± 0.15	27.67 ± 0.87	50.92 ± 1.21	75	3.49 ± 0.20	36.89 ± 1.16	67.89 ± 1.61
P 07^a^	2.28 ± 0.09	21.76 ± 0.97	44.07 ± 1.06	65	3.51 ± 0.13	33.48 ± 1.50	67.80 ± 1.64
P 08^a^	2.32 ± 0.15	27.10 ± 1.51	55.01 ± 1.66	85	2.73 ± 0.18	31.88 ± 1.78	64.72 ± 1.95
P 09^a^	2.98 ± 0.14	33.22 ± 0.95	62.70 ± 1.35	90	3.31 ± 0.16	36.91 ± 1.06	69.67 ± 1.50
P 10^a^	2.98 ± 0.14	20.87 ± 1.23	39.28 ± 0.43	56	5.32 ± 0.25	37.27 ± 2.19	70.14 ± 0.76
P 11^a^	2.88 ± 0.12	28.83 ± 0.84	55.64 ± 1.03	82.3	3.50 ± 0.15	35.01 ± 1.02	67.58 ± 1.25
Mean^b^	2.47	24.76	47.39	69.62	3.73	35.39	67.91
SD^b^	0.38	**6.03**	**10.55**	14.84	1.19	**2.72**	**2.38**

Note

Values are means ± SD. The bold values represent the standard deviations for individual times of DTh2 and DTh3. They clearly show that these inter‐individual differences were markedly smaller if DTh2 and DTh3 were expressed in relative terms (% *t*_max_) compared to absolute terms (min).

Abbreviations: DTh, duration threshold; Glu, blood glucose concentration; La, blood lactate concentration; RPE, rating of perceived exertion; SD, standard deviation; *t* abs., time expressed in absolute terms; *t* rel., time expressed in relative terms; *t*_max_, maximal time.

^a^ Values are averaged over all parameters except La, Glu, and RPE (*n* = 13).

^b^ Values are averaged over all parameters except La, Glu, and RPE as well as over all subjects (*n* = 11).

**FIGURE 7:**
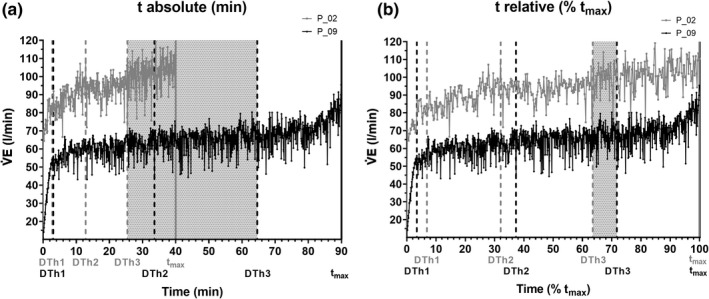
Times of DTh1, DTh2, DTh3, and *t*_max_ determined from individual V̇E curve patterns during the shortest (P 02) and the longest *t*_max_ CLT (P 09) expressed (a) in absolute (min) and (b) in relative terms (% *t*_max_). The gray areas represent the ranges for *t*_DTh3_ found between P 02 and P 09. The relative intensity was the same for both subjects. V̇E, ventilation; *t*, time; *t*_max_, maximal time; DTh, duration threshold; P, proband

DTh1, DTh2, and DTh3 as well as *t*_max_ detected from the individual curve patterns for V̇E of the subjects with the shortest (P 02) and the longest *t*_max_ CLT (P 09) are shown in Figure 7a (absolute terms: min) and Figure 7b (relative terms: % *t*_max_). For DTh3, the range between P 02 and P 09 was from 25.4 to 64.6 min (39.2 min) in absolute terms but only from 63.5 to 71.8% *t*_max_ (8.2% *t*_max_) in relative terms.

To show that the relative target intensity was the same for P 02 and P 09, it has to be mentioned that the V̇E values at DTh1, DTh2, and DTh3 were comparable between both subjects if they were expressed as % of V̇E at LTP_2_ from IET. Mean La und % HR_max_ values during the CLT were also comparable between these subjects.

## 4 DISCUSSION

The individualization of exercise prescription in training practice as well as in scientific studies is mainly based on exercise intensity but not on exercise duration. In our study, we could show that distinct duration domains and the according duration thresholds could be detected during a constant‐load test for the maximum duration (*t*_max_) as described by Platonov (1999). As there was a substantial interindividual difference in *t*_max_ despite the same relative exercise intensity, the second and third duration threshold (*t*_DTh2_, *t*_DTh3_) were also markedly different across subjects when they were expressed in absolute terms (min) but not in relative terms (% *t*_max_).

### 4.1 Interindividual differences in *t*_max_

This study clearly revealed that the maximal duration (*t*_max_ = “endurance capacity”) at a target workload of 10% *P*_max_ below P_LTP2_ showed a substantial interindividual difference with a range from 40 to 90 min although the group of subjects was rather homogeneous with respect to age and relative aerobic exercise performance (P_LTP1_, P_LTP2_, and P_max_). These data support the results of Moral‐Gonzalez et al. (2020) who also did not find a correlation between *t*_max_ and submaximal (respiratory compensation point) and maximal markers (*P*_max_) of aerobic fitness. Suggested main determinants of *t*_max_ are rather motion economy, the size of the glycogen stores, substrate utilization (oxidation of fatty acids vs. glucose), and the neuromuscular system's ability to work in fatigued conditions (Billat et al., 2001), where each of them may be associated with the training volume/week in individual training history. Therefore, the basis of an individualized exercise duration prescription is the determination of the *t*_max_ at given intensities by means of constant‐load tests until exhaustion (Mezzani et al., 2010, 2012).

The endurance capacity (Brooks et al., 2005) determined from a *t*_max_ CLT is—beside the power output at LTP_1_/VT_1_, LTP_2_/VT_2_, and *P*_max_ from an IET—an important component of one's endurance performance. For entire and individualized endurance performance diagnostics, we suggest the implementation of both IET and *t*_max_ CLT. However, any certain intensity has its own critical time limit. This fact is demonstrated by the power (or speed)—duration relationship which may be used as an individual diagnostic tool (Mezzani et al., 2010, 2012; Pettitt, 2016; Poole et al., 2016; Vanhatalo et al., 2011).

### 4.2 Distinct duration domains

Three distinct duration phases (1, 2 (a, b), and 3) separated by three duration thresholds were shown during the *t*_max_ CLT with significant differences in the slopes for 1 vs. 2a, 2a vs. 2b, and 2b vs. 3 found for V̇E, BF, EqO_2_, EQCO_2_, PETO_2_, and PETCO_2_ (Table 1). This is in accordance with the concept of Platonov (1999) who explained the distinction of two subphases within the phase of stable performance by an incomplete (2a) vs. complete stabilization (2b) of vegetative functions resulting in variations of physiologic responses and measures. This may be the reason why the curve shapes during 2a showed a further increase above (or decrease below) the value at target workload from IET, whereas stabilization of parameters was observed only during 2b. In our study, t_DTh3_ was concordant with Platonov's concept (60–75% *t*_max_) but not t_DTh2_ which was found at 30–40% *t*_max_ in our study vs. 40–60% *t*_max_ prescribed in Platonov's concept. Since our constant‐load tests were terminated when *t*_max_ was accomplished, the fourth phase of not‐compensated fatigue was not investigated in this study.

**FIGURE 8:**
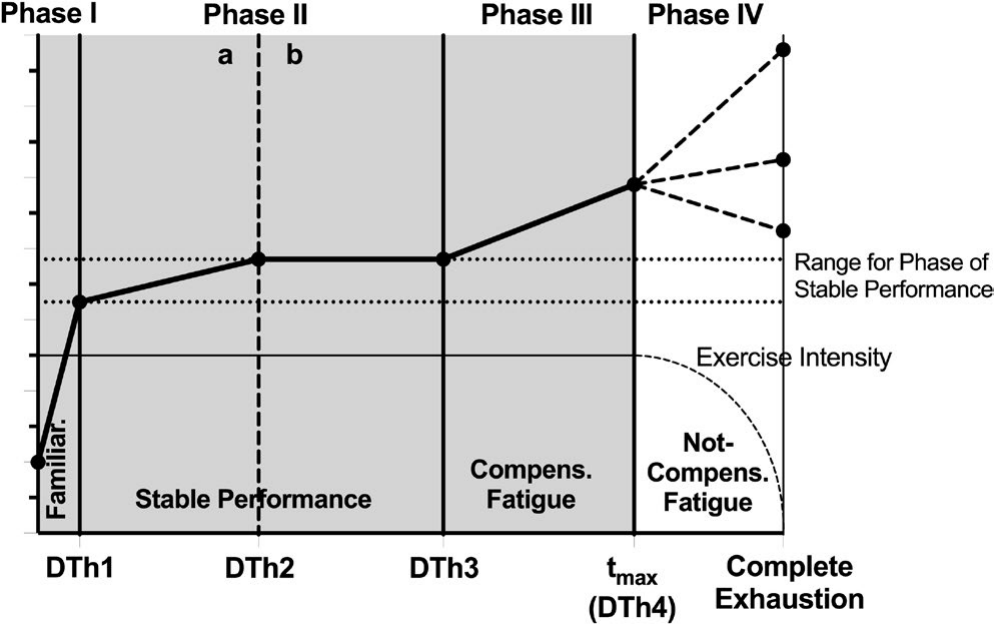
Schematic chart of distinct duration phases and duration thresholds during constant‐load exercise until complete exhaustion at a target intensity between P_LTP1_ and P_LTP2_ from IET. Curve shapes in phase 4 have not been investigated and are speculative. Familiar, familiarization; Compens., compensated; DTh, duration threshold; *t*_max_, maximal time

Based on our data and supporting the concept of Platonov (1999), the following schematic chart shows the four distinct duration phases and the according duration thresholds for physiologic parameters (except i.a. V̇T, PETCO_2_, and O_2_ pulse) during constant‐load exercise until complete exhaustion at a target intensity between P_LTP1/VT1_ and P_LTP2/VT2_ from IET (Figure 8).

At target intensities above *P*_LTP2_, physiologic parameters have been shown to increase continuously from DTh1 until *t*_max_ (Viru, 1995). At target intensities below *P*_LTP1/VT1_, *t*_max_ is considerably longer and, consequently, other limiting factors occur (Fasching et al., 2020; Pokan et al., 2014).

Interestingly, Hackney and Lane (2015) also described three distinct duration phases in their hormonal exercise response model (HERM). This is worth mentioning due to the critical role hormones are suggested to play in inducing acute physiologic responses and adjustments during exercise as well as training adaptations after exercise (Hackney, 2020; Hackney & Lane, 2015).

Our findings clearly point out that exercise duration matters. Besides intensity, also the duration of exercise (or rather the reached duration phase) is responsible for the acute physiologic responses to exercise. Importantly, exercise duration was also shown to have independent effects on training adaptations after training interventions (Hackney & Lane, 2015; Milesis et al., 1976; Platonov, 1999; Wenger & Bell, 1986).

### 4.3 Interindividual differences in duration thresholds (DTh) and *t*_max_ in absolute vs. relative terms

As described by Platonov (1999), the duration of the familiarization phase is suggested to depend on the applied intensity and type of warm‐up, but it is independent of *t*_max_. This was supported by our data which showed no correlation between *t*_DTh1_ and *t*_max_. Therefore, *t*_DTh1_ should be expressed in absolute terms (min). In contrast, the occurrence of DTh2 and DTh3 showed a highly significant correlation to *t*_max_. Since *t*_max_ varied widely across subjects, also *t*_DTh2_ and *t*_DTh3_ showed substantial interindividual differences if expressed in absolute terms. However, if *t*_DTh2_ and *t*_DTh3_ were expressed in relative terms (% *t*_max_), differences were significantly smaller across subjects (Table 2 and Figure 7).

These data illustrate the urgent need for an individualized prescription of exercise duration by means of % *t*_max_ or, even better, by % *t*_DTh2_ and *t*_DTh3_, corresponding to the individual prescription of exercise intensity by means of % LTP_1_/VT_1_ and LTP_2_/VT_2_. Although we just investigated one intensity, we suggest that knowing these duration thresholds enables a conscious and accurate regulation of exercise duration according to the training goal—improvement or stabilization of performance or even regeneration—for a certain intensity. As we did not investigate different intensities, it needs to be proven if this concept also applies to all other intensities also, as suggested by Platonov (1999). It allows every single person to exercise (1) long enough for gaining effectual training adaptations or (2) short enough either to avoid short‐term fatigue and overreaching as well as overtraining with undesirably long recovery periods or even to induce and accelerate recovery by the exercise of very short duration as proposed by Platonov (1999).

In contrast, exercise duration prescriptions via fixed absolute values, such as (20, 30, 45, 60, 90 min, etc. per session or per day), as usually applied in training practice and scientific studies (Diment et al., 2015; Karabulut et al., 2020; Myllymäki et al., 2012; Tremblay et al., 2005; Viru et al., 1996), may result in undesired and heterogeneous acute responses and training effects such as overreaching /overtraining or the nonresponder phenomenon (Lin et al., 2021; Ross et al., 2015), which makes it difficult or even impossible to compare studies. In addition, exercise with a too‐long total duration for a given intensity (near *t*_max_) may lead to health risks, particularly in patients (Mezzani et al., 2010, 2012). Therefore, especially when exercise sessions are too close together and regeneration is too short, common prescriptions for exercise duration should be scrutinized.

The required recovery time until the next exercise session with the same exercise type obviously depends on the combination of training duration and intensity and determines the planning of training sessions within a microcycle. This makes it even clearer how important it is to determine the duration phases individually.

Integrating the Turn Point Concept (Binder et al., 2008; Tschakert & Hofmann, 2013) for exercise intensity prescription and the duration threshold concept of the present study (Hofmann & Tschakert, 2017; Platonov, 1999) for exercise duration prescription into one combined model would allow an individualized exercise prescription. With respect to the planning of microcycles, the training frequency, which results from the recovery time, as well as the type of training must be additionally considered in accordance with the FITT principle (Burnet et al., 2019).

### 4.4 Limits of the study

In our pilot study, it was not possible to identify the reasons for the great diversity of *t*_max_ across subjects but we suggest that training volume (which was not obtained) may play a major role. However, our results are in line with those from Moral‐Gonzales et al. (2020) who presented a similar variability of *t*_max_.

The V̇O_2_ mean response time (MRT) (Iannetta et al., 2019) was not determined in this study. Differences in the MRT across subjects may have led to some imprecision in the determination of CLT target intensity, but we suggest only a minor impact in this regard. Small imprecisions in exercise prescription are unavoidable, if only because of possible differences in daily condition.

In our study, *t*_max_ constant‐load tests on a cycle ergometer were conducted at just one single exercise intensity (10% *P*_max_ below *P*_LTP2_). Therefore, it remains unclear if the findings of this study may be transferred to higher or lower intensities (and, consequently, varying duration domains), other endurance exercise modes such as running or cross‐country skiing, and for intermittent‐type exercise.

The group of subjects was homogenous with respect to age and relative exercise performance and all participants were moderately trained. Therefore, we cannot extrapolate the results of our pilot study to other populations such as patients, healthy sedentary people, or highly trained athletes. Furthermore, the proportion of males and females was unequal.

The nutritional status of the participants has not been obtained or controlled. It might be expected that the glycogen stores within muscles strongly influence *t*_max_. As subjects were instructed not to perform any strenuous exercise and to consume carbohydrate‐rich food before the *t*_max_ CLT, we do not expect a substantial influence in our study. However, these questions need to be addressed properly in well‐trained athletes with variable diets and exercise regimens such as taper periods.

There was no supply of water during the CLT. This might influence i.a. HR, cardiac output, thermoregulation, and, consequently, *t*_max_. Proving lower intensities and longer durations (*t*_max_) will require to supply food and water to the subjects (Pokan et al., 2014), but we suggest no substantial influence in our *t*_max_ CLT.

### 4.5 Future perspectives

It needs to be investigated if the duration domains and thresholds revealed in this study are also valid for other intensity domains (below LTP_1_/VT_1_, between LTP_1_/VT_1_ and LTP_2_/VT_2_, and above LTP_2_/VT_2_), other parameters such as hormones, immune parameters, etc., varying subject groups, and different exercise modes. In addition, training intervention studies investigating the duration‐dependent training adaptations by using individualized exercise duration prescriptions are urgently needed comparable to resistance training studies investigating all‐out exercise to failure compared with submaximal repetition numbers (Vieira et al., 2019, 2021).

Since the rating of perceived exertion showed distinct duration phases and thresholds in our study and marked drifts at certain times (comparable to DTh3) in other studies (Green et al., 2009; Lajoie et al., 2000), the question arises whether RPE can be used as a practicable training tool to control and regulate exercise duration.

## 5 CONCLUSION

This study clearly revealed a possible critical role of exercise duration regarding the acute physiologic responses to exercise. The duration of the two subphases of stable performance and of the phase of compensated fatigue as well as the according duration thresholds (*t*_DTh2_, *t*_DTh3_) were significantly related to the maximum duration (*t*_max_) which was observed to be markedly different across subjects. Consequently, also *t*_DTh2_ and *t*_DTh3_ showed a substantial interindividual difference when they were expressed in absolute terms. Therefore, it is recommended to prescribe exercise duration on an individual basis by means of relative terms such as % *t*_max_ or % DT.

## CONFLICT OF INTEREST

None of the authors has any conflicts of interest, financial, or otherwise to disclose.

## AUTHOR CONTRIBUTION

GT and PH conceived and designed research; GT, TH, LW, PB, AM, and WG performed experiments; GT, TH, and LW analyzed data; GT and PH interpreted results of experiments; GT prepared figures; GT drafted manuscript; GT and PH edited and revised the manuscript; GT and PH approved final version of the manuscript.
